# Supplementary material for: Overview of Meta-Analyses: The Impact of Dietary Lifestyle on Stroke Risk
Source: Int J Environ Res Public Health. 2019 Sep 25;16(19):3582. doi: 10.3390/ijerph16193582 (PMC6801861; doi:10.3390/ijerph16193582)
Supplement: Supplementary file 1 [file ijerph-16-03582-s001.zip › ijerph-582977-Supplementary Table S2.pdf]

Table S2. Summary of dose response analysis in studies considered

Author	Food or beverage intake	Control intake	Type of strokes	Evaluated dose for each food or beverage	Number of primary studies	Effects size (95% C.I.)
COHORT STUDIES						
**Alexander 2015 [5]**	Dairy	No	Ischemic or Hemorrhagic	<1.5 serving/day	-	0.92 (0.89; 0.96)
				≥1.5 serving/day	-	0.91 (0.88; 0.95)
	Milk	No		0-1 serving/day	-	0.95 (0.86; 1.04)
				>1 to <2 serving/day	-	0.98 (0.90; 1.06)
				≥2 serving/day	-	1.01 (0.92; 1.11)
	Cheese	No		0-0.5 serving/day	-	1.00 (0.92; 1.07)
				>0.5-1.5 serving/day	-	0.86 (0.75; 0.97)
				>1.5 serving/day	-	0.92 (0.87; 0.97)
	Dairy and Ca	No		0-100 mg/d Ca from dairy product	-	0.91 (0.84; 1.00)
				>100-300 mg/d Ca from dairy product	-	0.67 (0.58; 0.77)
				>300 mg/d Ca from dairy product	-	0.82 (0.69; 0.97)
**Larsson 2016 [12]**	Alcohol consumption	Non-drinkers; never drinkers; occasional drinkers.	Ischemic	<1 drink/day	20	0.90 (0.85; 0.95)
				1-2 drink/day	20	0.92 (0.87; 0.97)
				2-4 drink/day	21	1.08 (1.01; 1.15)
				>4 drink/day	12	1.14 (1.02;1.28)
			Hemorrhagic	<1 drink/day	9	0.92 (0.77; 1.10)
				1-2 drink/day	8	0.99 (0.82; 1.18)
				2-4 drink/day	8	1.25 (0.93; 1.67)
				>4 drink/day	8	1.67 (1.25; 2.23)
			Subarachnoid Hemorrhage	<1 drink/day	9	1.21 (0.96; 1.52)
				1-2 drink/day	6	1.11 (0.80; 1.53)
				2-4 drink/day	9	1.39 (0.94;.2.07)
				>4 drink/day	8	1.82 (1.18; 2.82)
**Aune 2017 [19]**	Fruit and vegetables	No	Ischemic or Hemorrhagic	200 g/day	15	
						0.92 (0.90; 0.94)
	Fruit	No		200 g/day	24	0.90 (0.86; 0.94)
	Vegetables	No		200 g/day	20	0.84 (0.79; 0.90)
	Apples, pears	No		100 g/day	5	0.94 (0.84; 1.05)

	Berries	No		100 g/day	5	1.07 (0.79; 1.45)
	Citrus Fruits	No		100 g/day	9	0.78 (0.69; 0.90)
	Citrus Fruits juice	No		100 g/day	2	0.89 (0.72; 1.10)
	Dried fruits	No		100 g/day	1	0.75 (0.32; 1.81)
	Fruits juice	No		100 g/day	2	0.72 (0.63; 0.83)
	Grapes	No		100 g/day	2	0.57 (0.34; 0.97)
	Allium vegetables	No		100 g/day	1	0.89 (0.76; 1.04)
	Cruciferous vegetables	No		100 g/day	5	1.04 (0.80; 1.36)
	Green leafy vegetables	No		100 g/day	5	0.73 (0.57; 0.94)
	Pickled vegetables	No		100 g/day	2	0.57 (0.43; 0.74)
	Potatoes	No		100 g/day	4	0.98 (0.94; 1.02)
	Root vegetables	No		100 g/day	2	0.96 (0.78; 1.18)
	Tomatoes	No		100 g/day	4	1.01 (0.96; 1.06)
	Berries	No	Ischemic	100 g/day	3	1.02 (0.61; 1.72)
	Citrus fruits	No		100 g/day	7	0.87 (0.79; 0.95)
	Citrus Fruits juice	No		100 g/day	2	0.87 (0.80; 0.96)
	Allium vegetables	No		100 g/day	2	0.93 (0.77; 1.11)
	Cruciferous vegetables	No		100 g/day	5	0.66 (0.41; 1.07)
	Green leafy vegetables	No		100 g/day	4	0.74 (0.62; 0.89)
	Potatoes	No		100 g/day	5	1.00 (0.95; 1.05)
	Root vegetables	No		100 g/day	3	0.91 (0.64; 1.30)
	Tomatoes	No		100 g/day	2	0.92 (0.86; 0.98)
	Berries	No	Hemorrhagic	100 g/day	3	1.66 (0.91; 3.03)
	Citrus fruits	No		100 g/day	3	0.79 (0.59; 1.06)
	Cruciferous vegetables	No		100 g/day	2	0,27 (0.01; 12.54)
	Potatoes	No		100 g/day	3	1.03 (0.91; 1.16)
	Root vegetables	No		100 g/day	2	1.16 (0.66; 2.02)
**Aune 2016 [30]**	High Whole grain bread	Low intake Whole grain bread	Ischemic or Hemorrhagic	90 g/day	1	0.88 (0.72; 1.07)
	High intake Whole grain breakfast cereals	Low intake Whole grain breakfast cereals		30 g/day	2	1.07 (0.69; 1.64)
	High intake refined grain	Low intake refined grain		90 g/day	5	0.91 (0.81; 1.02)
	High intake total rice	Low intake total rice		100 g/day	4	1.00 (0.97; 1.03)
	High intake total grains	Low intake total grains		90 g/day	5	0.93 (0.85; 1.02)

**Chen [53]**	C vitamin		Ischemic or Hemorrhagic	Incremental 100 mg/day	10	0.83 (0.75; 0.93)
**Tang [52]**	Flavoids		Ischemic or Hemorrhagic	Incremental 100 mg/day	3	0.91 (0.77; 1.08)
**RCT**						
**Tian 2017 [27]**	FA*** supplementation	No supplementation	Ischemic or hemorrhagic	<2 mg	-	0.78 (0.68; 0.89)
	FA supplementation	No supplementation		≥2 mg	-	0.96 (0.88; 1.04)
	Daily Vit B12	No supplementation		<0.5 mg	-	0.93 (0.78; 1.10)
	Daily Vit B12	No supplementation		≥0.5 mg	-	0.94 (0.86; 1.03)

*** Folic Acid
